# Hosts for Hostile Protein Production: The Challenge of Recombinant Immunotoxin Expression

**DOI:** 10.3390/biomedicines7020038

**Published:** 2019-05-17

**Authors:** Stefania Zuppone, Maria Serena Fabbrini, Riccardo Vago

**Affiliations:** 1 Urological Research Institute, Division of Experimental Oncology, IRCCS San Raffaele Hospital, 20132 Milan, Italy; zuppone.stefania@hsr.it; 2 MIUR, Italian Ministry of Instruction, University and Research, 20090 Monza, Italy; msfabbrini@gmail.com; 3 Università Vita-Salute San Raffaele, 23132 Milan, Italy

**Keywords:** recombinant immunotoxin, bacterial/eukaryotic expression systems, ribosome inactivating proteins, toxin-based drugs

## Abstract

For the recombinant expression of toxin-based drugs, a crucial step lies not only in the choice of the production host(s) but also in the accurate design of the protein chimera. These issues are particularly important since such products may be toxic to the expressing host itself. To avoid or limit the toxicity to productive cells while obtaining a consistent yield in chimeric protein, several systems from bacterial to mammalian host cells have been employed. In this review, we will discuss the development of immunotoxin (IT) expression, placing special emphasis on advantages and on potential drawbacks, as one single perfect host for every chimeric protein toxin or ligand does not exist.

## 1. Introduction

Targeted therapy aims to specifically attack diseased cells while leaving healthy cells unaffected. This kind of treatment has been especially, but not exclusively, utilized in the treatment of tumours, where chemotherapy approaches or radiation therapy may cause severe side effects through the impact on rapidly dividing cells in blood, the digestive system and hair follicles, thus inducing blood disorders, nausea and vomiting and, less serious but still psychologically impactful, hair loss. “Denileukin diftitox” (Ontak^®^) was the first FDA-approved toxin-based formulation employed in clinical approaches for the treatment of cutaneous T-cell lymphomas [1]. Ontak^®^ is a recombinant fusion protein consisting of human interleukin-2 (IL-2) conjugated to diphtheria toxin (DT) fragments A and B, which include the enzymatically active site and the membrane translocation domain. It is expressed using a bacterial system. Ontak^®^ was approved for the treatment of CD25 positive cutaneous T-cell lymphoma expressing IL-2 receptor alpha (IL2RA). The expression of this receptor allows for internalization of the drug, followed by escape from the endomembrane system resulting in cell death [2]. Recently, a second-generation IL-2 receptor-targeted diphtheria fusion toxin was produced and it was demonstrated to exert antitumor activity alone and in combination with anti–PD1 in melanoma [3].

Toxin-based drugs are most effective against haematological tumours as they can be directly injected into the bloodstream and can thus easily reach the transformed target cells. Originally, the so-called immunotoxins (ITs) consisted of an intact monoclonal antibody domain chemically conjugated to a toxic domain and were directed towards lymphoma and leukaemia specific markers such as CD3, CD19, CD20, CD22, CD30 and CD38 [4]. Solid tumours could also be treated with ITs, albeit showing less efficacy likely due to the partial immunotoxin (IT) penetration within the tumour mass. In fact, the therapeutic success of any tumour targeting agent is strictly correlated with its successful delivery to the tumour site at a sufficient concentration with an uniform distribution throughout the neoplastic lesion(s) [5]. In addition, the effectiveness may also be reduced due to the onset of immunogenicity, either to the toxin domain or to the whole monoclonal antibodies used [6]. Two main strategies can be used to manage this drawback: the first one is local administration to bypass the immune system, which, however, has limited utility for oncology; the second one is the deimmunization of the immunotoxin. Point mutations techniques have been widely used in order to replace critical immunoreactive amino acids in both the antibody [7,8,9] and the toxic payload portions. In 1994, Behnar at al. deimmunized the B3 Fv antibody portion of a PE38 based immunotoxin (B3(Fv)-PE38) by “framework exchange,” which involves the substitution of murine residues with human ones [8]. A truncated form of diphtheria-toxin (DT390) was also deimmunized by the mutation of highly hydrophilic R, K, D, E and Q amino acids, which were located in protruding positions on the molecule surface, away from the catalytic site. The toxin mutant was demonstrated to lose only a minimal activity in in vitro cytotoxic assays, with a significant 90% reduction of anti-toxin antibodies in toxin-immunized mice [10]. Similarly, Onda et al. produced a less immunogenic-PE38 based immunotoxin by replacing large hydrophilic amino acids—recognized as B cell epitopes—on PE38 [8]. The mutated product showed full cytotoxic and antitumor activity with a lower immunogenicity demonstrated in three strains of mice [8]. Later on, LBM11, an IT combining an anti-CD22 Fab with a less immunogenic version of PE38 was developed, where most of domain II containing the B and T cell epitopes was deleted and 7-point mutations in domain III were inserted to suppress B cell epitopes. LMB11was tolerated at much higher doses in mice than wild type IT and treatment resulted in a complete remission of the Burkitt Lymphoma in this mouse model [11].

The plant derived type I ribosome inactivating protein (RIP) bouganin was successfully deimmunized in three specific epitopes recognized by a T cell assay performed using PBMCs from 20 donors. The T-cell epitope-depleted variant was genetically linked to an anti-epithelial cell adhesion molecule (EpCAM) Fab moiety to create the fusion construct VB6-845, which selectively killed EpCAM-positive cell lines [12]. In vivo efficacy was demonstrated using a human ovarian tumour xenograft model with most mice treated being tumour free at the end of the study [12]. Other plant RIPs, such as trichosanthin [13] and alpha-momorcharin [14], were also studied and modified to reduce their immunogenic potential. Possible immunogenic sites in the saporin sequence were predicted by comparing conserved portions in its structure to the other above-mentioned three Type I RIPs so far analysed [9]. A similar strategy has been adopted with previously identified toxin residues found to cause vascular leak syndrome (VLS) via unspecific binding to vascular endothelial cells: mutations in a single amino acid flanking the consensus sequence responsible for VLS displayed a significant reduction in vascular damage by ricin A chain (RTA) [15]. An analogous approach was carried out also for Pseudomonas Exotoxin A (PEA) [16] and DT [3], where engineered toxins were proven to retain potent anti-tumour activity but with a remarkable attenuation of VLS.

## 2. Immunotoxin Development

**Figure 1:**
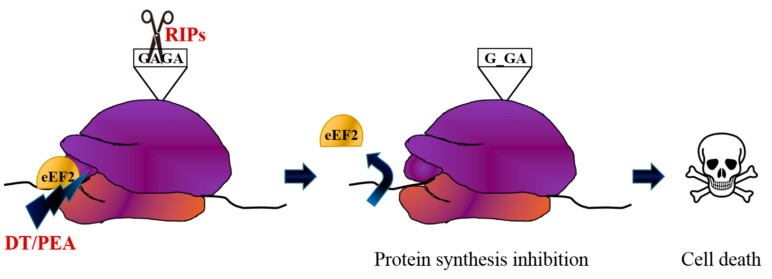
Toxin mechanisms of action. Ribosome inactivating proteins (RIPs) such as ricin and saporin depurinate a specific adenine in a universally conserved GAGA-tetraloop in the rRNA, while diphtheria toxin (DT) and pseudomonas exotoxin A (PEA) inactivate the eukaryotic elongation factor 2 (eEF2) by ADP ribosylation, both causing protein synthesis inhibition and thus cell death.

The first attempt to selectively deliver a toxin to cancerous cells were made by chemically conjugating a toxin domain to an antibody, which confers the specificity to the IT target. Bacterial or plant toxins have been employed for this purpose: the bacterial toxins Pseudomonas Exotoxin A (PEA) and DT together with the plant RIPs ricin and saporin have been most frequently studied for therapeutic purposes but several others are under evaluation, predominantly in the oncological field. [17]. Toxins are powerful, natural weapons that have increased in their toxicity by the pressure of natural selection over millions of years and subsequently only a tiny number of molecules are needed to exert overwhelming effects. PEA and DT directly inactivate EF-2 by ADP ribosylation, thereby inhibiting amino acid chain elongation during protein synthesis. Ricin, saporin and other RIPs, such as pokeweed antiviral protein (PAP), gelonin, bouganin and trichosanthin, depurinate a specific adenine base located in the universally conserved GAGA-tetraloop, present in the 23/26/28S ribosomal RNA (rRNA) (Figure 1). A few of these toxins are active on bacteria, yeast, plants and animals - virtually every living thing. The final effect is a consequence of the irreversible blocking of protein synthesis, which in turn causes cell death. While the type II RIP ricin is formed by a catalytic domain bound to a binding domain, saporin and others type I RIPs lacking the latter, comprise a sole effector moiety with a toxic power hundreds of folds lower. It was soon clear that the binding domain of the type II RIP was hazardous and should be inactivated or eliminated. In fact, the cell binding moiety of ricin, the B chain, is a lectin, which is able to recognize galactose residues, driving the catalytic active A domain inside virtually every type of cell [18,19].

The use of protein toxins is particularly appealing due to the high intrinsic activity of the catalytic domain; the cell cycle-independent mechanism of action, which allows for the killing of non-actively duplicating tumour cells; and the escape of common multidrug resistance pathways.

**Figure 2:**
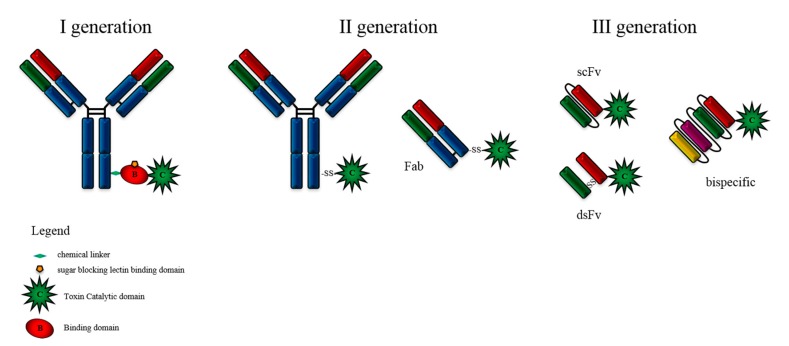
Development of immunotoxins for targeted therapy. Three generations of immunotoxins (Its) are displayed. First generation ITs were prepared by chemically conjugating antibodies to intact toxin with attenuated cell binding capability. In the second generation ITs, truncated toxins lacking binding domain were conjugated to the antibody antigen-binding fragment (Fab). Third generation ITs were produced through genetic engineering and the targeting moieties are represented by the light and heavy chain variable fragments either genetically linked (single-chain variable fragment—scFv) or held together by a disulphide bond (dsFv). Bispecific ITs contain two monodomains with different specificities.

The first generation of ITs were prepared by using the whole toxin with attenuated cell binding capability, which was chemically linked to the antibody. The obtained product was greatly heterogeneous in its composition and lacked stability, causing high variability among batches. Other concerns arose from the high immunogenicity, safety issues and high costs of production under GMP conditions. Reducible linkers were introduced to facilitate the detachment of the active domain from the antibody, therefore allowing its translocation to the cytosol. Elucidation of the crystal structure of various toxins enabled significant improvements in IT design, leading to the second generation, where only the catalytic domain of the toxin was conjugated to the antibody or to an antigen-binding fragment (Fab). The latter enabled an improved tumour penetration due to its reduced size compared to the full-length antibodies. Several ITs demonstrated high activity and specificity and were tested in evaluated in phase I trials in cancer patients. However, the cost of production was prohibitive and the product was still chemically heterogeneous. The third generation, mostly produced in bacteria, is represented by wholly recombinant molecules that have overcome past limitations by containing only the peptide domains needed to target and kill tumour cells. This was achieved by replacing the cell-binding domain with a ligand or with the light and heavy chain variable fragments of an antibody, either genetically linked (scFv) or held together by a disulphide bond (dsFv) (Figure 2). Targeting domains might also be further modified to increase their cellular specificity and binding affinity. Such recombinant ITs are homogeneous and much less expensive to produce [19,20,21].

The advantages of recombinant ITs over those chemically conjugated to antibody domains include: 1. the reduced size, which increases the penetration capacity into the solid tumour environment; 2. the one-step production process leads to a more homogeneous batch compared to the two-step protein production followed by chemical conjugation; 3. the management and scaling of productions easier, with a subsequent reduction of the manufacturing costs and quicker production combined with adequate yields of the therapeutic molecule. However, the chemical conjugation process involving an antibody and a toxin domain initially requires the production of each component independently, which can therefore be carried out in different appropriate hosts, according to the structural properties of the proteins. As a result, a more precise and effective folding can be achieved for both domains, retaining their native structure.

## 3. Immunotoxin Factories

Most commercial production of recombinant proteins for therapeutic purpose involves the use of bacteria, yeast or mammalian cell lines as expression hosts. The identification of the best host cell to produce the protein of interest is the first and, in some ways, the most important step that will initiate and drive the outline of the whole process. No ideal organism able to efficiently and safely produce all kinds of protein toxic chimerae is available, so the choice of the expression system has to be carefully weighted depending on the final product. It is worth mentioning the intrinsic paradox in the production of ITs, which can poison the host cells. Therefore, in identifying the most suitable expression system, it is necessary to consider the possible autointoxication.

Bacteria were the first engineered organism able to produce exogenous proteins [22] and this system has continued to be improved. It may be suitable to produce proteins that do not contain complex post-translational modifications. However, the folding machinery of prokaryotic cells is often not proficient in producing fully functional folded proteins of heterologous origin. Commonly, partially folded proteins are confined to insoluble inclusion bodies and have to be extracted and refolded to be active. These laborious processes are time consuming and can determine a reduction of the final yield. Multiple strategies, including temperature reduction and optimization of induction conditions, are employed to reduce the expression rate and better couple it to the protein folding. Alternative strategies comprise the co-expression of chaperone complexes, belonging to the protein quality control system, assisting nascent polypeptides to reach their native structure [23]. A main concern in the employment of bacterial expression systems during the production of biopharmaceutical drugs is that of endotoxin contamination. Endotoxins can form stable interactions with other biomolecules thus making their removal difficult after protein purification. Even if present in small quantities, they can cause fever, inflammation, sepsis and tissue damage and even lead to death [24].

Eukaryotic protein expression systems, including yeast, insect and mammalian cells developed more recently, allow for the addition of complex post-translational modifications (e.g., *N*-glycosylation) and may regulate protein activity, stability and interactions with partner molecules [25,26]. For pharmaceutical purposes, recombinant expression in microorganisms is generally preferred, as bacteria and yeast both offer a cost-effective high level of protein expression, fast cell growth, simple media requirement and lower costs. In addition, a wide range of plasmids resulting in multiple combinations of replicons, promoters, selection markers, multiple cloning sites and fusion protein strategies, is available [27]. A significant proportion of eukaryotic proteins (around 50%) is glycosylated and thus necessitates the introduction of *N*-glycans or more complex sugar modifications which is undertaken in the endoplasmic reticulum (ER) and Golgi-complex by specific modifying enzymes [28], essential for the production of Fc fusion protein drugs [29]. Proteolytic cleavage, phosphorylation, acetylation and methylation are some examples of the modifications that are necessary for proteins to achieve their native and thus functional, form, that can be better provided by eukaryotic cells [30].

More recently, eukaryotic microalgae have been proposed as platforms for light-driven synthesis of recombinant proteins. The production has been established in algal chloroplasts - organelles containing a minimal genome and therefore suitable for rapid engineering to allow high-level, regulated and stable expression of the transgene. A clear advantage resides in the encapsulation into chloroplasts allowing the accumulation of protein toxins otherwise dangerous for the host [31].

### 3.1. Bacteria

Bacteria grow in rich complex media readily available and made from inexpensive components. They can be transformed with exogenous plasmid DNA in a fast and easy manner [27]. The expression of recombinant proteins within *E. coli* can be affected by several factors, including plasmid copy number, mRNA stability, upstream elements required for efficient transcription, growth time, temperature and codon usage. To date, toxins and ITs are mainly produced in bacterial and yeast host cells [32,33]. One common problem to overcome during their production resides in their intrinsic toxicity toward the host ribosomes. A few RIPs kill cells by affecting targets that are conserved from bacteria and yeast to plants and animals. First efforts to express recombinant toxins in *E. coli* were very challenging, because, upon toxin induction, the bacterial growth rate was significantly compromised due to depurination of *E. coli* ribosomes [34,35,36]. To limit this deleterious effect, the expression has to be tightly regulated and some *E. coli* mutants were specifically selected to withstand the expression of toxic proteins. Specialized *E. coli* strains, for example BL21(DE3)pLysS, allow inducible gene expression, while suppressing the basal expression of the protein, to avoid autointoxication [37,38]. The λDE3 lysogen carries the gene for T7 RNA polymerase under the control of the lacUV5 promoter. The target gene can be expressed only in the presence of T7 RNA polymerase, which is induced by Isopropyl β-D-1-thiogalactopyranoside (IPTG), an analogue of lactose. The pLysS encodes T7 lysozyme, which lowers the background expression level of target genes under the control of the T7 promoter but does not interfere with the level of expression achieved upon IPTG induction. Recombinant molecules, where toxic domains were fused to the variable fragment (Fv) of a mAb [39] or more recently to single-chain Fv (scFv), which consists of the Fv heavy and light chain fragments covalently connected with a flexible polypeptide linker sequence, were inserted upstream or downstream of the catalytic domain of the toxins [33]. To further improve the stability of recombinant ITs, disulphide-stabilized Fv (dsFv) molecules were successively developed [40].

Another critical issue in the exogenous production is the efficiency of protein codon recognition among different organisms. When the frequency of synonymous codons in the exogenous DNA is substantially different from that of the host, a codon bias can occur. Consequently, a depletion of low-abundance tRNAs takes place during the synthesis of the recombinant protein, causing amino acid misincorporation and/or premature truncation of the polypeptide, resulting in a reduction of the protein expression levels [41]. Upon detecting the presence of rare codons in a given gene when *E. coli* is used as a host, codon optimization of the sequence improves the production yield without affecting the protein activity [42]. So far, a considerable number of ITs have been produced in bacteria and demonstrated to be active and specific in various preclinical models. For instance, the recombinant IT D2C7-(scdsFv)-PE38KDEL, specific for both wild-type epidermal growth factor receptor (EGFR) and for its deletion mutant EGFRvIII exhibited potent antineoplastic effects against intracranial glioblastoma xenografts [43]. PEA-based recombinant immunotoxins anti-Tac(Fv)-PE38, targeting CD25 and RFB4(dsFv)-PE38 (also known as BL22), targeting CD22, have each been tested in patients with interesting results. In a phase I trial anti-Tac(Fv)-PE38 IT displayed clinical activity in CD25-positive hematologic malignancies and was relatively non-immunogenic [44]. A phase II trial conducted with BL22 confirmed the activity of the IT in chemoresistant hairy cell leukaemia [45]. To improve the affinity of BL22 for CD22, phage selection was used to identify random mutations in the CDR3 domain of VH. A three amino acid mutant showed 14-fold enhanced binding affinity due to lower off-rate [46]. The resulting disulphide-stabilized recombinant IT—named moxetumomab pasudotox—was tested in preclinical models, showing similar toxicology and improved cytotoxicity compared to BL22 [47]. A phase I clinical trial was conducted with moxetumomab pasudotox in relapsed/refractory hairy cell leukaemia patients, showing a positive safety profile [48] and is now undergoing multicentre phase III testing [49].

Recent advanced in the field are demonstrated by SL401, a recombinant fusion protein composed of the catalytic and translocation domains of DT fused to IL-3, which is produced in *E. coli* and selective for IL-3 receptor positive human myeloid leukaemia cell lines [50]. SL401 was demonstrated to be effective (including complete remission) in blastic plasmacytoid dendritic cell neoplasm (BPDCN) and in other haematological malignancies. [51,52]. Multiple phase I/II clinical trials have been conducted using this drug, achieving encouraging results, and SL401 has been just approved by the FDA for adult and paediatric BPDCN [53].

### 3.2. Intracellular Immunization and Issues Related to Host Auto-Intoxication

Host auto-intoxication was observed when we first expressed a secretory chimera preATF-saporin in an eukaryotic expression system, as well as more recently expressing the precursor of a seed saporin isoform in Tobacco Protoplast [54]. We showed in this case that, by using specific anti-saporin neutralizing IgGs, we could protect the wheat germ ribosomes from intoxication, thus obtaining the expression of wild type saporin in vitro. This demonstrated that ribosome protection was indeed due to the concomitant presence of the immune Igs in the assay.

“Cytosolic immunization” was first used in the title of our FASEB J. Manuscript to indicate the exploitation of neutralizing anti-saporin antibodies that were co-microinjected into the *Xenopus oocyte* cytosol to allow for expression of a chimera between N-terminal fragment of human urokinase and seed saporin isoform (preATF-SAP mRNA, which was co-injected). Co-injection of control IgG did not result in any expression of the chimera [55]. This strategy allowed the production of a highly toxic secretory protein in eukaryotic cells, avoiding cell suicide caused by autointoxication. The procedure consisted of equipping host cells with cytosolic neutralizing antibodies directed toward the toxic domain of the heterologous polypeptide and this intracellular immunization was found to be essential for the synthesis of correctly folded, biologically active ATF-SAP in the high amounts needed to investigate its in vivo anti-metastatic potential. For secreting a toxic chimera like the one described above, we would need to have a mammalian cell system stably expressing in the cytosolic compartment the variable domains essential to neutralize the tiny amounts of newly synthesized toxic polypeptide that would escape ER-segregation. Single domain antibodies (sdAbs) are nowadays available from camels or sharks, being comprised only of the variable heavy chain domain. These “sdAbs” seem to be very stable, nonaggregating molecules as compared to whole antibodies or to single chain Fv fragments, being potentially the best suited novel inhibitors of cytosolic proteins [56].

At that time (2000) we had, however, no such novel strategy in our hands and the principal idea deriving from the intracellular immunization approach described in the *X. oocyte* expression system was planning to obtain a “protected” universal eukaryotic host, exploiting a CHO cell line, that was already under investigation in our lab [57], able to perform complex *N*-glycosylation patterns. Normally, CHO cells lack the Golgi enzyme, α-2,6-sialyltransferase (α-2,6-SiaT) but are able to add core structures of O-glycosylation found in human proteins (a modification also present in the human ATF domain); for a comprehensive review on CHO glycosylation patterns please refer to Reference [58]. In addition, the α-2,6-SiaT cells were also able to grow in suspension in fermenters, allowing for the purification of secreted therapeutic molecules from the conditioned media.

Stably transformed CHO alpha2,6-sialyltransferase cells should have ideally expressed in the cytosol the saporin neutralizing variable fragments to avoid host auto-intoxication. In order to achieve this, we first planned to adopt library-panning procedures in order to identify potentially neutralizing single chain antibodies against saporin in collaboration with E. Benvenuto’s laboratory in Enea, where panning procedures and single chain scFv libraries were widely used. Surprisingly, the panning procedure was deleterious when using seed-extracted saporin loaded on the column where the phage library was being passed: instead of selectively enriching phages, we observed a gradual and constant depletion in phages, suggesting a possible intoxication of the bacterial layer by the immobilized toxin (A. Desiderio; R. Ippoliti and MS Fabbrini, unpublished observations). We later explored the *Pichia pastoris* platform where PAP and DT-containing chimaeras could be successfully expressed and secreted by these eukaryotic microbial hosts [59,60]. However, even in this eukaryotic system we observed some toxicity-related issues.

When initially transforming with pPicSAPWT *Pichia pastoris* yeast cells, we observed that only very few colonies with unusual phenotypes could be obtained (morphology was disrupted with the colonies, instead of being round, were a sort of “meringue” in shape (A. Lombardi and MS Fabbrini, unpublished observation), while parallel transformations with a pPicSAPKQ saporin catalytic-site mutant gave rise to an expected number of transformed (round-shaped) colonies. We reasoned that such an event could be due to truncated polypeptides or translation-arrested partial saporin-derived peptides that were still able to intoxicate/interfere with the yeast host cell. This preliminary observation prompted us to synthesize a yeast-optimized saporin gene version for further investigation, which gave the positive expected results [61]. We therefore adopted the strategy of codon-optimization with all of the subsequent constructs we expressed in *P. pastoris* to avoid interruption or stress-related events during the exogenous protein translation [33,62].

Based on the afore-mentioned assumption, even just a single catalytic active site/polypeptide can irreversibly harm the host cell. Therefore, successful toxin or chimaera expression requires the absence of quality control by resident chaperones in the ER, which would otherwise lead to the undesired retro-translocation of the toxic nascent protein/chimaera polypeptides to the cytosol for degradation. Instead, we should try our best to favour an efficient and smooth passage along the secretory route to achieve extracellular secretion of the toxic polypeptides. In fact, if the scFv or the targeting domain have intrinsic folding problems (as we have experienced with our synthetic anti-CD22 scFv, [33] this may raise the kind of issues that we first reported and discussed above. These parameters are important to keep in mind when choosing the most effective signal peptide for insertion into the ER—secretory route of a toxic chimaera or when designing the optimal linker peptides that join the different domains (both those linking the heavy and light variable antibody chains, as well as the precise order—either VH-linker-VL or VL-linker-VH). In this regard, we showed that the native signal peptide of saporin behaves as a stress-sensor, favouring its translocation to the cytosol, when we expressed precursor saporin polypeptides in plant Tobacco protoplasts [54], whereas the signal peptide of ER-resident chaperones, such as binding immunoglobulin protein (BiP) or protein disulphide isomerase (PDI), having quite different biochemical properties [18] may behave in the exact opposite way and may therefore represent an option to consider. Synthetic assembled domains (as in the case of scFv) could indeed show folding problems tending to form protein aggregates. We have constructed and tested ten different alternative chimeric fusions that were expressed in *P. pastoris*: the best option for the antiCD22scFv domain was the anti-CD22 RB4 fused to PE38 with a (G_4_S)_3_ linker between the VH-VL chains [33]. This type of strategy should be explored when designing a toxic protein chimaera with several alternative choices to be systematically assayed in order to find the best performing one, not only for the yeast expressed proteins but also for the expression of PE38-based IT.

### 3.3. Yeast

Eukaryotic systems present several advantages compared with bacteria, including the possibility of the recombinant protein being secreted into the culture media and of effecting complex post-translational modifications. However, the main drawbacks of these organisms are the higher cost, laborious management of cultures and time-consuming processes of production. Yeast maintains the advantages of unicellular organisms in terms of cell cultures combining them to specialized management of protein production. The methylotrophic yeast *Pichia pastoris* has been largely used for the production of heterologous proteins since it allows only secretion-competent polypeptides to reach the extracellular medium by assuring a proper oxidative folding process [63,64]. This system is particularly suitable for the expression of certain toxins such as mellitin [65], diphtheria toxin, killer toxin or saporin [61,66], since heterologous protein production can be induced by switching the carbon source from glycerol/glucose to methanol when high biomass has been achieved and toxic proteins can be rapidly and efficiently sorted in the secretion pathway and secreted. The safety of this expression system was confirmed for saporin by demonstrating that the expression of a catalytically inactive saporin KQ mutant displayed the same growth rate and production yield than for the wild type toxin [62]. The GS115 yeast strain was found to be particularly tolerant to the expression of bacterial toxins and the recombinant, CD3-targeting Diphtheria toxin-based immunotoxin fusion was one of the first successfully expressed up to 10mg/L [60]. One important issue being the insertion into the endomembrane system and for this purpose an efficient signal peptide must be used to avoid auto-intoxication problems [18]. Other toxins or toxin-based products were designed and efficiently expressed in *P. pastoris* yeast, displaying catalytic activity comparable to wild type proteins. For instance, recombinant PAP-I produced in yeast demonstrated the same enzymatic activity of native PAP extracted from *Phytolacca americana* L. Host toxicity was avoided through rapid and efficient secretion of the toxin into the culture medium [59]. SAP and SAP chimera production in *P. pastoris* was improved following codon-usage optimization, which greatly increased expression levels with the product as cytotoxic as the seed-extracted protein [61]. Monovalent, bivalent and single-chain fold-back diabody anti-human CCR4 DT-based immunotoxins were successfully produced in yeast and demonstrated to be effective and specific in a human CCR4^+^ tumour bearing *NSG* mouse model [67].

In order to select the best microbial system for expression of a chimeric scFv-toxin, we have prepared two constructs expressing the anti-CD22 scFv fused to either PEA or SAP and compared their expression in *E. coli* or *P. pastoris* [33]. Bacteria were faster in producing the ITs but they had to be extracted from inclusion bodies and renatured, while yeast cells necessitated a longer time for the production phase but the secreted ITs were fully functional after an easier purification step. The ITs containing a toxin moiety of bacterial origin are better expressed in the *E. coli* host, while saporin-based ITs are best expressed in the *P. pastoris* system. Notably, the activity of the resulting ITs was comparable on Burkitt’s lymphoma cells overexpressing the CD22 receptor [33]. This result corroborates the assumption that a unique host for the expression of all the toxins does not exist. Proteolysis may occur at different levels of the yeast secretory pathway, during vesicular transport by resident proteases or in the extracellular space by secreted or cell wall-associated proteases. Additionally, during high cell density culture proteases can be released in the supernatant as a result of cell disruption [63]. To tackle proteolysis problems, multiple strategies have been tested by modifying each of the following: fermentation parameters (pH, temperature and growth rate), the media composition (addition of amino acids or peptone, reduction of salt concentration), the application of protein engineering strategies to remove critical sequences and engineering the expression host to obtain protease-deficient strains [68]. Every variation in the toxin sequence can induce a conformational modification thus affecting the catalytic activity and has to be carefully evaluated. The use of protease-deficient strains for the expression of protease-sensitive proteins has been reported for at least two decades with heterogeneous results. The principal reason is that in several cases, more than one protease can be involved in the degradation process and, hence, it becomes very challenging to optimize the expression of a heterologous protein by knocking out just a single hydrolytic activity.

### 3.4. Plant Cells

To prevent self-intoxication of producing cells, most of native plants synthesize RIPs as inactive precursor proteins and store them in vacuoles. In this way, the mechanism of toxin biosynthesis ensures that enzyme and substrates never encounter, leaving overall protein synthesis unaffected [69]. The idea to use plant cells to produce RIPs was realized by using plant tobacco protoplasts. Preproricin was successfully expressed in tobacco protoplasts and its processing and targeting of the vacuole occurred efficiently, avoiding any toxicity for the host, while the expression of RTA is retro-translocated to the cytosol followed by protein synthesis inhibition [70]. The type I precursor protein preprotrichosanthin was properly processed in tobacco protoplasts and the final protein product showed toxic activity similar to the native one only when the precursor included the C-terminal propeptide [71], indicating an efficient ER-segregation signal for this RIP or that the C-terminal propeptide did interfere with its toxicity. However, the saporin precursor was not efficiently expressed in tobacco protoplasts and thus an incomplete ER translocation of the nascent polypeptide precursor due to an ER-stress response could be the reason for this observation. The selection of ER signal peptides, for example chaperones such as PDI or BIP, may be an important criterion to allow a proper folding and an efficient ER-segregation of toxic protein chimeras including saporin [54].

Recently, eukaryotic green microalgae have been explored as a potential platform for the production of complex therapeutic and industrially relevant recombinant proteins. To date, more than 20 therapeutically important proteins such as vaccines, human antibodies and ITs [72] have been successfully expressed in this unicellular green biosystem, mainly in *Chlamydomonas reinhardtii* [73]. The use of unicellular algae as cell factories represents, a low-cost, low-tech and sustainable approach, especially for countries that cannot have fermentation infrastructures. Their high growth rate (their biomass doubles within 24 h and a very short period of time is required to scale up the initial microalgal transformant volumes needed for large scale production), ease of cultivation (which can be carried out phototrophically or heterotrophically in photobioreactors, where parameters such as light intensity (2500–5000 lux), temperature (18–24 °C), pH (8.2–8.7), nutrient quantity, carbon dioxide (1.85 g CO_2_/g biomass or higher) can be monitored and controlled) lead to both improving the management of the bioprocess and ensuring a robust final yield of the product of interest. In addition, many green algae are edible and algal species for the food ingredients and health food markets already have the GRAS (generally recognized as safe) status. This provides the possibility to explore the oral delivery of bioactive proteins, so avoiding costly investment in the purification process [31]. The use of green microalgae for heterologous proteins production has many advantages over bacteria, including the ability of the former organisms to perform complex post translational modifications like glycosylation and to produce secreted proteins [74,75,76].

The diatom *P. tricornutum*, for instance, was used to produce a monoclonal antibody directed against Hepatitis B virus surface protein. The heavy and light chain were successfully expressed, retained in the endoplasmic reticulum by DDEL retention peptides and shown to be correctly assembled and glycosylated [77]. One year later, the same group demonstrated that omitting the ER retention signal from the constructs, the antibody was similarly correctly folded and secreted in the culture medium in the active form [78].

Another important feature that encourages the use of green microalgae as expression system is the availability of different transformation methodologies for nuclear, chloroplast and mitochondrial genomes, which results in the quick generation of positive transformants [79]. A potential drawback of nuclear expression is characterized by proteolytic degradation into the cytosol. Proteolytic enzymes are essential for endogenous protein processing and removal of defective or abnormal native proteins. For nuclear-expression, proteins synthesis can be targeted to the ER by adding an H/KDEL C-terminal tetrapeptide tag in the construct [80,81]. This strategy leads to decrease the cytosolic proteolytic degradation of the product and to a final yield, which is generally 2-fold to 10-fold higher [80]. Another possible strategy to minimize proteolytic degradation of proteins requiring post-translational modification could consist of targeting the nuclear-expressed proteins to the chloroplast for accumulation and storage [82].

The majority of the research in this field has focused on chloroplast genome manipulation for expressing and assembling complex heterologous proteins, even though it results in a lower productivity compared to the nuclear genes and it lacks a post translational modification system (e.g., glycosylation) [83,84]. This organelle contains a minimal polyploid genome, deriving from a cyanobacterial progenitor that mostly encodes for core components of the photosynthetic complexes and the chloroplast’s transcription/translation apparatus. It is characterized by a circular molecule of 200 kb, which is present in roughly 80 identical copies, all of them needed to be converted in the recombinant form. Since plastid transformation is achieved through homologous recombination, transgenes can be precisely targeted to specific genomic loci and regulated [85,86]. In order to optimize the protein production machinery, many different steps affecting chloroplast gene expression and translation should be considered in the genetic engineering process. Among these, the most relevant are transcription, mRNA processing, mRNA splicing, mRNA stability, initiation of translation and protein turnover which are regulated by nuclear-encoded factors, often gene specific [87,88,89,90,91,92,93,94]. Indeed, some studies demonstrated that 5 ′UTRs of plastid mRNAs contain key elements for translational regulation [95,96,97,98]. In 2005, Barnes et al. fused different promoters and 5′ (*atpA*, *rbcL*, *psbA*, *psbD* and *16S rRNA)* and 3′ (*atpA*, *rbcL*, *psbA* or *tRNA _ar_*_g_) UTR regions of Chlamydomonas chloroplast genes to a GFP reporter gene, obtaining different fusion products which were integrated in the chloroplast genome [99]. These fusions resulted in the proportional accumulation of chimeric mRNA and proteins at very different levels, with a peak for fusions carrying atpA or psbD promoters and 5′UTRs. A very low protein accumulation was detected under control of *rbcL* and *psbA*, in contrast to 16S rRNA 5′UTR, which did not induce any at all [99]. PsbA and atpA chloroplast promoters were also used to support the expression of three recombinant proteins such as 14FN3, VEGF and HMGB1 [100]. Interestingly, the psbA promoter along with the corresponding UTRs allowed the achievement of a 20-fold increase in protein accumulation with respect to constructs carrying the atpA promoter and its 5′UTR. Algal derived VEGF was found to have a dose dependent binding activity to its receptor, with a slightly lower affinity compared to its bacterial derived homologue, probably due to the presence of misfolded or truncated VEGF in the assayed samples [100].

Unlike bacteria, chloroplasts contain a wide range of chaperones and folding enzymes that allow them to correctly fold the complex proteins of the photosynthetic apparatus as well as recombinant proteins, which accumulate as soluble and functional molecules within the chloroplast [84]. However, although the chloroplast does not possess the machinery for protein glycosylation, it is capable of correctly folding and assembling antibodies for which glycosylation is not essential to bind their target [101]. Transgenic strains producing either the monovalent or divalent CD22-targeted, gelonin-based ITs have been developed and demonstrated to express soluble, enzymatically active products capable of specific binding to target cancer B-cells and reducing their viability [102]. Not only plant RIPs but also bacterial toxins were selected as catalytic domain for the IT production in the same context. Anti-CD22-PEA ITs were efficiently expressed and correctly folded in chloroplasts of microalgae. Both monovalent or divalent ITs induced apoptotic cell death in Burkitt’s lymphoma cells and significantly inhibited tumour growth in a xenograft mouse model, improving animal survival rate [103].

### 3.5. Toxin-Resistant Cells

Liu and co-workers explored the possibility of specifically engineering eukaryotic cells in order to create DT resistant strains able to improve the production yield of DT-based products [104]. In particular, they used for the first time a DT-resistant CHO cell line to produce a monovalent, truncated anti-T cell immunotoxin, DT390-scFvUCHT1. The mutation was achieved using chemical mutagenesis by substituting an arginine to glycine at position 701 of EF-2 encoding gene, making *EF-2* non-ADP-ribosylatable by DT or PEA [104]. The resulting CHO-K1 RC1.22c mutant cell line has been successfully used for expression of the immunotoxin [105,106] achieving a level of 4 µg/mL active product secreted into the medium. A few years later, the same group focused on the production of the bivalent anti-T cell immunotoxin A-dmDT390-bisFv(G4S), a multi-domain protein containing the catalytic (A chain) and translocation domains of DT and four Fv domains (VL and VH) of the anti-CD3 antibody UCHT1, highly effective in depleting T cells in the treatment of T cells leukaemia. For this purpose, given the advantage of *P. pastoris* in the expression of heterologous proteins, they generated a DT- resistant strain by inserting a single substitution of arginine for glycine, resulting in a resistance to ADP-ribosylation of EF-2 exerted by the toxin [104]. The IT expression by the secretory route was able to achieve up to 10–35 mg/L in shake flask or bioreactor culture, respectively 2- to 7-fold higher than what could be attained by CHO cells [104,105]. Furthermore, EF-2 yeast mutants were found to be highly resistant to DT-A chain, showing an increased viability compared to WT strains. However, the amount of immunotoxin produced was not augmented but generated more degradation products that were found both in cell medium and pellet, most likely to be caused by intracellular proteolysis, since the medium derived immunotoxin exhibited stability both in the presence and absence of cells. In this work, they also demonstrated that the secretory machinery of the expressing cells can produce only a limited amount of the heterologous product, as suggested by the fact that a double copy expression resulted in an increased amount of the truncated form of the IT, suggesting that the excess material was degraded.

## 4. Concluding Remarks

Toxins have been studied for decades and have been exploited as potent and versatile weapons against cancers or other human diseases. In this context, ITs were developed as soon as monoclonal antibodies were inserted in the concept of targeted therapy, characterized by specificity and limited adverse effects. Increasing knowledge about toxins’ structure, properties, intoxication route and mechanism of action enabled the conversion of a lethal molecule into a therapeutic agent. Codon optimization, removal of potential antigenic epitopes combined with the possibility to employ humanized antibody domains have led to a significant improvement of IT expression and application. IT expression has been investigated in multiple organisms, including bacteria, yeast and plant cells. As recombinant proteins’ therapeutic efficiency mostly relies on their correct folding, eukaryotic hosts are preferable to bacteria. In fact, even if the advantage of microbial expression platforms is the cost-effectiveness together with their easy scaling up process for industrial production, complex organisms are more appropriate production platforms as they are able to guarantee a well assembled final product. Chinese hamster ovary (CHO) cells, for instance, have been widely used to this purpose, mainly due to their glycosylation machinery, which is very similar to that of human cells [58,106]. Of relevance is an artificial CHO cell line, called ‘universal host’ (UH CHO), engineered to express a rat derived alpha2,6-sialyltransferase (EC 2.4.99.1;K2,6-ST), thus able to introduce N terminal sialic acid residues to both K2,3- and K2,6-linkages of glycans of human origin [57]. This acquired ability has led to the production of interferon (IFN) gamma-based recombinant proteins with improved pharmacokinetics compared to IFN-gamma secreted by regular CHO cells. However, improving protein yield is one of the first and foremost goals and it is the prerequisite to scale up the production. Genetic engineering of the hosts allowed for the selection of appropriate strains to tolerate high amount of ITs, without being intoxicated [104,105]. The further improvement of heterologous expression hosts coupled with cost-effective production is likely to give a substantial contribution in terms of efficacy and safety, making ITs suitable for clinical applications mainly in combination with surgery, chemo- or radiotherapy.
